# MicroRNA-214 Inhibits Chicken Myoblasts Proliferation, Promotes Their Differentiation, and Targets the TRMT61A Gene

**DOI:** 10.3390/genes11121400

**Published:** 2020-11-25

**Authors:** Yanjun Duan, Yulin Wu, Xuemei Yin, Tingting Li, Fuxiang Chen, Pengfei Wu, Shanshan Zhang, Jinyu Wang, Genxi Zhang

**Affiliations:** College of Animal Science and Technology, Yangzhou University, Yangzhou 225000, China; yjduan1996@163.com (Y.D.); ylwu1995@126.com (Y.W.); xmyin1990@163.com (X.Y.); d150070@yzu.edu.cn (T.L.); fxchen1993@163.com (F.C.); wu_p_fei@163.com (P.W.); ZSSzzz12345@163.com (S.Z.); gxzhang@yzu.edu.cn (G.Z.)

**Keywords:** myoblast, miR-214, proliferation, differentiation, Dual-Luciferase Reporter assay

## Abstract

The proliferation and differentiation of myoblasts is an important process of skeletal muscle development. In this process, microRNAs (miRNAs) play an important role in the proliferation and differentiation of chicken primary myoblasts (CPMs). Our previous study found that miR-214 and the tRNA methyltransferase 61A (TRMT61A) gene were differentially expressed in different stages of proliferation and differentiation. Therefore, this study aimed to explore the effect of miR-214 on the proliferation and differentiation of CPMs and the functional relationship between miR-214 and TRMT61A. In this study, we detected the effect of miR-214 on the proliferation of CPMs by qPCR, flow cytometry, CCK-8, and EdU after the overexpression and interference of miR-214. qPCR, Western blotting, and indirect immunofluorescence were used to detect the effect of miR-214 on the differentiation of the CPMs. The expression patterns of miR-214 and TRMT61A were observed at different time points of differentiation induced by the CPMs. The results show that miR-214 inhibited the proliferation of the CPMs and promoted the differentiation of the CPMs. The Dual-Luciferase Reporter assay and the expression pattern of miR-214 and TRMT61A suggested that they had a negative regulatory target relationship. This study revealed the function and regulatory mechanism of miR-214 in the proliferation and differentiation of CPMs.

## 1. Introduction

Skeletal muscle is an important part of the body of livestock and poultry, accounting for 40–60% of the total body weight. Skeletal muscle participates in the regulation of animal movement, health, metabolism, and so on [1], and a decrease in muscle content causes problems such as a higher incidence rate, lower motor ability, and higher mortality in animals. A study of muscle growth and development can provide important guidance for the production performance of livestock and poultry. Muscle fiber is an important part of skeletal muscle, and the difference in the number of muscle fibers affects muscle production [2]. In animal genomes, microRNAs (miRNAs) are involved in the regulation of 30% of gene expression [3]. Many miRNAs have been proven to be involved in the regulation of skeletal muscle growth and development. miR-203 can participate in the proliferation and differentiation of chicken primary myoblasts (CPMs) and muscle development [4]. miR-223 affects the proliferation and differentiation of CPMs by dynamically regulating the expression of its target genes, thus affecting muscle development [5]. Therefore, the role of miRNAs in skeletal muscle development should not be ignored.

At present, reports about miR-214 mostly concern the regulation of various cancer cells. miR-214 is highly expressed in gastric cancer cells, and the knockout of miR-214 can inhibit the migration, proliferation, and invasion of gastric cancer cells through the PTEN gene-mediated signal pathway [6]. miR-214 can inhibit the development of hepatocellular carcinoma (HCC) by regulating the expression of zeste homologue 2 (EZH2), β-catenin (ctnb1), and E-cadherin (CDH1) [7]. In addition, miR-214 can accurately induce muscle cell types, and the inhibition of miR-214 leads to a decrease in or loss of slow muscle cell types [8]. miR-214 can also act on mammalian mouse C2C12 myoblasts and can participate in the mitosis and myogenic differentiation of mouse C2C12 myoblasts [9]. In our previous study [10], we found that miR-214 was differentially expressed during the proliferation and differentiation of CPMs. However, the effect of miR-214 on the proliferation and differentiation of CPMs has not yet been reported.

In our previous study, the tRNA methyltransferase 61A (TRMT61A) gene was also found to be differentially expressed during the proliferation and differentiation of CPMs_._ We combined with the miRDB database (http://www.miRdb.org/miRDB/) and found that TRMT61A may be a potential target of miR-214. TRMT61A is located on chromosome 5 of the chicken genome. It can catalyze m^1^A, which is a rare modification in the cytoplasm, and it can provide new research direction in post-transcriptional regulation [11]. At present, the biological function of TRMT61A in myoblasts has not been reported.

The growth of embryonic skeletal muscle mainly depends on the proliferation and differentiation of myoblasts. In this study, the effects of miR-214 on the proliferation and differentiation of CPMs and the functional relationship between miR-214 and TRMT61A were explored.

## 2. Materials and Methods

### 2.1. Ethical Statement

This experiment was approved by the Animal Welfare Committee of Yangzhou University (license no.: syxk (Su) 2012-0029) and was conducted according to the Guidelines of Animal Use of the Committee of Ministry of Agriculture of China (Beijing, China). All efforts were made to minimize the suffering of the animals.

### 2.2. Animals and RNA Extraction

Haiyang yellow chicken eggs were obtained from Jiangsu Jinghai Poultry Industry Group and incubated at 55–60% humidity and 37.5 °C. Three female chicken embryos of 12 embryo age (E12), of 14 embryo age (E14), of 16 embryo age (E16), of 18 embryo age (E18), and 1 day old (D1) were selected, and the Trizol method was used to extract RNA. The NanoDrop-1000 Micro Nucleic Acid Analyzer (Thermo, Waltham, MA, USA) was used to detect the concentration and purity of RNA, which was then stored at −80 °C for later use.

### 2.3. cDNA Synthesis and Design of the Primers for Quantitative Real-Time PCR (qPCR)

Reverse transcription was carried out by using the HiScript QRT SuperMix for qPCR (+gDNA wiper) Kit (Vazyme, Nanjing, China) according to the manufacturer’s instructions, and miRNA was performed using the miRNA 1st-strand cDNA Synthesis Kit (by stem loop). The reverse transcription primer and internal reference primer U6 of miRNA were designed according to the software provided by the company manufacturer (Vazyme, Nanjing, China). Gene primers were designed using the Ensembl database and Primer Premier 5.0 software (PREMIER Biosoft International, Palo Alto, CA, USA). The p21, MyHC, MyoG, and MyoD primers were from references in the work of Cai et al. [12]. qPCR of mRNA was performed using the ChamQ SYBR qPCR Master Mix Kit (Vazyme, Nanjing, China). miRNA was quantified using the Universal SYBR qPCR Master Mix Kit (Vazyme, Nanjing, China). Steps for the qPCR experiment were provided by the supplier. The gene primer sequence-related information is shown in Table 1, while the related primer sequence information in the miR-214 quantitative experiment is shown in Table 2. Lastly, the sequence information of miR-214 overexpression, interference, and negative control are displayed in Table 3.

### 2.4. Isolation and Culture of CPMs

Muscle tissue was isolated from the leg muscles of the E12 chickens, chopped with scissors, washed with Hanks balance solution (Solarbio, Beijing, China), and then digested with 0.2% type I collagenase (Solarbio, Beijing, China) in a 37 °C water bath for 20 min. After that, the cells were collected by 1500 r centrifugation, and the filtrate was discarded. The cells were resuspended in DME/F-12 1:1 (1×) medium (Sigma Aldrich, St. Louis, MO, USA) with 20% fetal bovine serum and 1% penicillin/streptomycin/gentamicin mixed solution (Solarbio, Beijing, China). At 37 °C for 40 min, the fibroblasts were removed to enrich the CPMs, and the process was repeated twice. Then, 20% fetal bovine serum (FBS) medium was used under normal conditions. During differentiation, the 20% FBS medium was replaced by 2% horse serum medium in an aseptic incubator at 37 °C with 5% CO_2_.

### 2.5. CCK-8 Assay

The cells were seeded on a 96-well plate, transfected with the Cell Counting Kit-8 (Dojindo, Kumamoto, Japan), and then the absorbance was detected at 450 nm at 12 h, 24 h, 36 h, and 48 h.

### 2.6. Cell Cycle Analysis

When the density of the primary myoblasts reached approximately 60%, they were transfected with miR-214 mimic, mimic NC, miR-214 inhibitor, and inhibitor NC, with three replicates in each group. After 24 h, the solution was changed. When the cell density reached approximately 90%, the cells were collected with trypsin and centrifuged to remove the supernatant. The cells were reconstituted with pre-cooled PBS, added to with pre-cooled absolute ethanol, and fixed overnight at −20 °C. After fixation, the supernatant was removed by centrifugation, and then the supernatant was centrifuged by pre-cooling PBS suspension cells. At the end of fixation, PBS and RNase a solution (Sangon Biotechnology, Shanghai, China) were added, evenly blown, and placed at 37 °C for 20 min. Afterward, a propidium iodide (PI) staining solution (Solarbio, Beijing, China) was added, followed by light avoidance at 4 °C for 30 min, and then tested on a machine. Flow cytometric analysis was performed on a FACSAria SORP flow cytometer (BD Company, Franklin, NJ, USA), and Modfit LT software 4.0 was used to process the data.

### 2.7. EdU Assay

The cells were seeded on a 24-well plate. The transfection of miR-214 mimic, mimic NC, miR-214 inhibitor, and inhibitor NC was started when the cell density was 60%. After 48 h, the cells were fixed and stained with the EdU Detection Kit (Ribobio, Guangzhou, China). Three different fields of view were randomly photographed in each group with a fluorescent inverted microscope, and statistical analysis was performed with image-pro Plus 6.0. The proliferation rates were calculated as EdU-positive cells/Hoechst-stained cells, and the group with the lower proliferation rate was adjusted to 1.

### 2.8. Construction and Transfection of Plasmid

The 3′UTR region of TRMT61A was amplified by PCR from the cDNA of chicken leg muscles and cloned into the pMIR-REPORT™ miRNA Expression Reporter Vector System (Thermo Fisher, Shanghai, China). The mutant vector was constructed by site-directed mutagenesis with Mut Express MultiS Fast Mutagenesis Kit V2 (Vazyme, Nanjing, China). Cctgctg was mutated into aagtagt.

293T cells were used for the Dual-Luciferase Reporter assay. After co-transfection, for the TRMT61A-3′UTR-WT (wild-type) or TRMT61A-3′UTR-MT (mutant-type) plasmids with the miR-214 mimic or mimic NC, the firefly and Renilla luciferase activities were measured at 48 h post-transfection using a Dual-Luciferase Reporter Assay Kit (Vazyme, Nanjing, China) according to the manufacturer’s instructions. Luminescence was measured using a full wavelength microplate reader (Perkin Elmer, Waltham, MA, USA) and the firefly luciferase activities were normalized to Renilla luminescence in each well.

### 2.9. Immunofluorescence

After the induction of CPMs for 72 h, they were fixed with 4% paraformaldehyde (Solarbio, Beijing, China) for 30 min, and washed with PBS 3 times, 5 min each time. Afterward, 0.3% Triton X-100 (Solarbio, Beijing, China) was infiltrated for 15 min, and blocked with normal goat serum (Solarbio, Beijing, China) for 30 min at 37 °C. Rabbit Anti-Desmin Polyclonal Antibody (1:500; BIOSS, Beijing, China) was incubated overnight at 4 °C, while DyLight 594 combined affinity Goat Anti-Rabbit IgG (H+L) (1:300; Boster Bio, Wuhan, China) was incubated for 1 h at 37 °C. Then, images were taken under a fluorescence-inverted microscope. Three different fields of vision were randomly selected from each group and statistically analyzed with Image Pro Plus 6.0.

### 2.10. Western Blotting Assay

Cellular protein was extracted using radio immune precipitation assay (RIPA) buffer (Beyotime, Shanghai, China) with phenylmethanesulfonyl fluoride (PMSF) protease inhibitor (Beyotime, China). After incubation on ice for 10 min, the supernatant was collected by centrifugation at 10,000× *g* for 5 min at 4 °C. Then the protein concentration was determined to keep the final concentration of each group of samples as consistent as possible. The protein samples were electrophoresed and transferred to the membrane, and then the standard procedures were used for protein detection. The antibodies used were as follows: MYH1 Rabbit Polyclonal Antibody (1:500; Group Proteintech, Wuhan, China), Anti-GAPDH Antibody (1:3000; Huabio, Hangzhou, China), and HRP-Conjugated Goat Anti-Rabbit IgG (1:5000; BBI, Shanghai, China).

### 2.11. Statistical Analysis

Data are presented as mean ± SEM of at least three independent experiments. SPSS 19.0 software (SPSS Inc., Chicago, IL, USA) was used for statistical analysis. The unpaired Student’s *t*-test was used to compare the two groups. *p* < 0.05 (*) or *p* < 0.01 (**) indicates that the data are statistically significant.

## 3. Results

### 3.1. miR-214 Inhibits the Proliferation of Chicken Myoblasts

**Figure 1:**
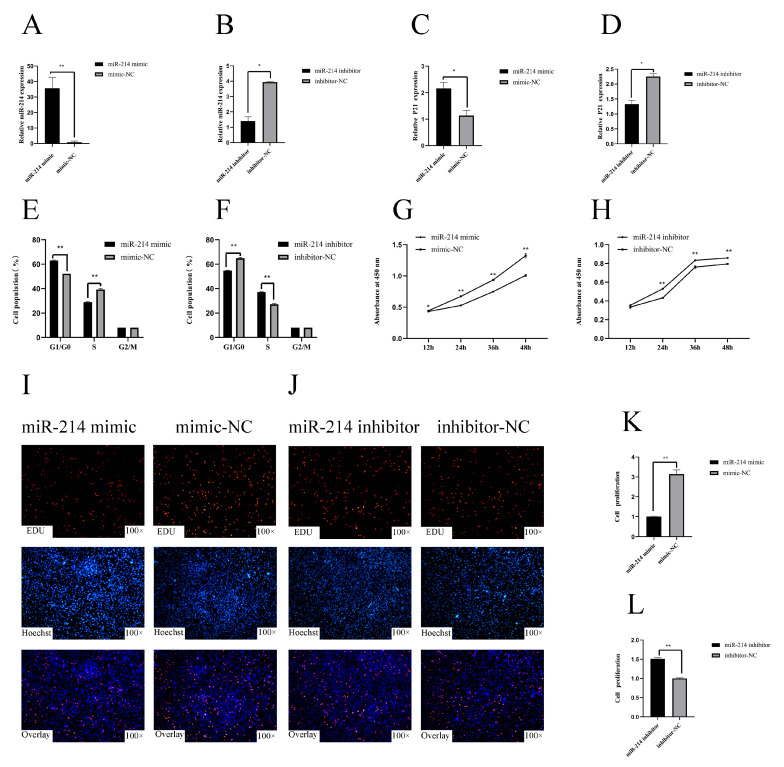
miR-214 inhibits the proliferation of chicken myoblasts. (**A**,**B**) The relative mRNA expression of miR-214 after transfection with the miR-214 mimic and inhibitor in chicken primary myoblasts (CPMs). (**C**,**D**) The relative mRNA expression of p21 after transfection with the miR-214 mimic and inhibitor in CPMs. (**E**,**F**) Cell cycle analysis of the CPMs 48 h after the overexpression and inhibition of miR-214 using propidium iodide staining for DNA content. (**G**,**H**) Cell growth was measured following transfection of the miR-214 mimic and inhibitor in CPMs. (**I**,**J**) After miR-214 overexpression and interference, EdU detected the proliferation of CPMs. (**K**,**L**) Proliferation rates of CPMs with miR-214 overexpression and inhibition. * *p* < 0.05 and ** *p* < 0.01.

In order to reveal the function of miR-214, we overexpressed and interfered with miR-214, and detected the relative expression of miR-214 in CPMs after 48 h (Figure 1A,B). In the chicken myoblasts, the overexpression of miR-214 significantly increased the expression of the p21 gene, increased the number of cells in the G0/G1 phase, and decreased the number of cells in the S phase (Figure 1C,E). On the contrary, miR-214 inhibition significantly reduced the relative expression of the p21 gene, decreased the number of cells in the G0/G1 phase, and increased the number of cells in the S phase (Figure 1D,F). We also found that the overexpression of miR-214 inhibited the proliferation of myoblasts (Figure 1G,I,K), whereas the inhibition of miR-214 enhanced the proliferation of the myoblasts (Figure 1H,J,L). These results suggest that miR-214 can inhibit the proliferation of CPMs.

### 3.2. miR-214 Promotes the Differentiation of Chicken Myoblasts

**Figure 2:**
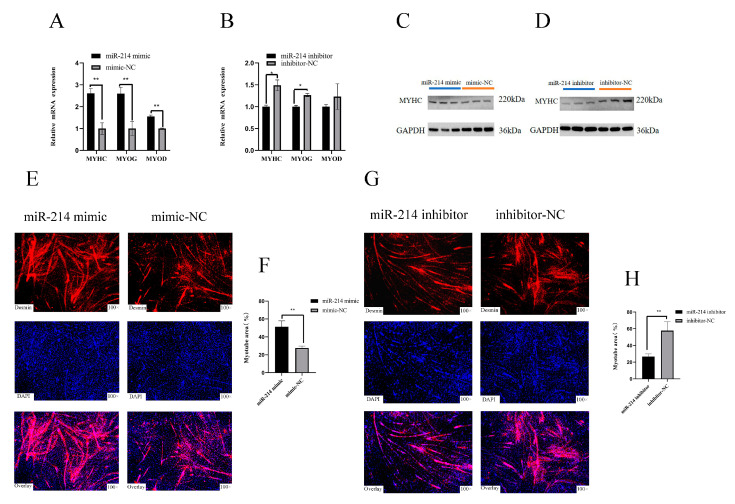
miR-214 promotes the differentiation of chicken myoblasts. (**A**,**B**) The relative mRNA expression levels of MYHC, MYOG, and MYOD after miR-214 overexpression and interference. (**C**,**D**) The expression of MYHC protein after miR-214 overexpression and interference. (**E**,**G**) CPMs stained with Desmin 72 h after miR-214 overexpression and interference. (**F**,**H**) The proportion of myotube area 72 h after miR-214 overexpression and interference. * p < 0.05 and ** *p* < 0.01.

In order to further study the function of miR-214, we induced the differentiation of CPMs in vitro and overexpressed and interfered with miR-214. After 72 h of overexpression of miR-214, the mRNA expression levels of the differentiation marker genes MyHC, MyoD, and MyoG in the CPMs were significantly increased. On the contrary, after transfection with the miR-214 inhibitor, the relative expression levels of MyHC and MyoG were significantly decreased (Figure 2A,B). After miR-214 overexpression, the protein level of MyHC was significantly increased. On the contrary, after interfering with miR-214, the protein level of MyHC was significantly reduced (Figure 2C,D). Indirect immunofluorescence showed that the overexpression of miR-214 significantly increased the total myotube area (Figure 2E,F), whereas miR-214 inhibition decreased the myotube area (Figure 2G,H). These findings suggest that miR-214 can promote the differentiation of CPMs.

### 3.3. miR-214 Targets TRMT61A and Negatively Regulates TRMT61A

**Figure 3:**
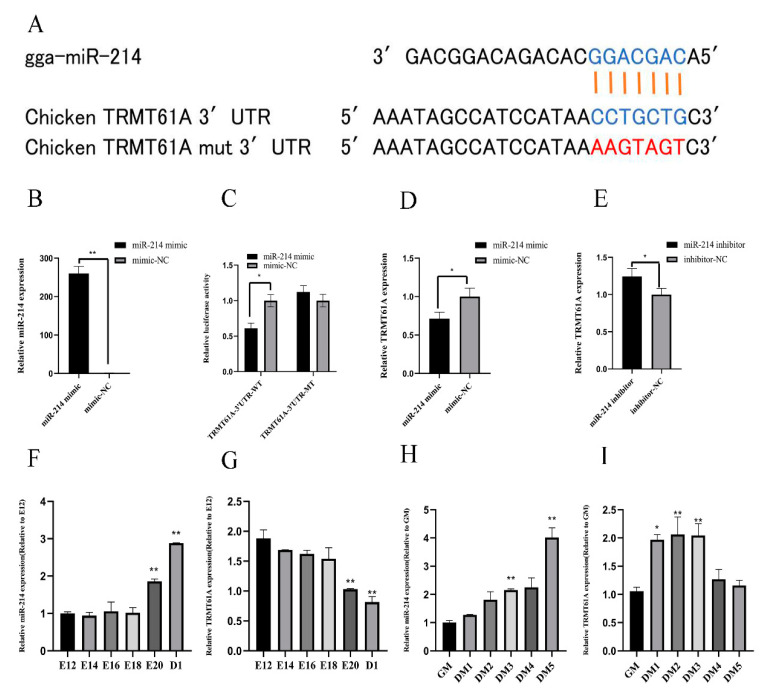
miR-214 targets TRMT61A and negatively regulates TRMT61A. (**A**) The potential binding site of miR-214 in the TRMT61A mRNA 3′UTR. The mutation sequence for the miR-214 binding site is shown in red. (**B**) The relative expression of miR-214 after transfection of the miR-214 mimic into 293T cells. (**C**) A luciferase assay was conducted by co-transfecting wild-type or mutant TRMT61A 3′UTR with the miR-214 mimic or the mimic NC in 293T cells. (**D**,**E**) The expression level of TRMT61A in the CPMs after miR-214 overexpression and interference. (**F**,**G**) The expression pattern of miR-214 and TRMT61A in the leg muscle in the embryonic period and at one day old. (**H**,**I**) The expression patterns of miR-214 and TRMT61A during the period of proliferation and differentiation. * *p* < 0.05 and ** *p* < 0.01.

We used qPCR to detect the relative expression of miR-214 and TRMT61A from E12 to D1, and found that miR-214 showed an upward trend, while TRMT61A showed a downward trend (Figure 3F,G). We induced the differentiation of CPMs in vitro for five days. As the induced differentiation progressed, miR-214 showed a gradual upward trend, while TRMT61A showed a gradual downward trend (Figure 3H,I). We speculate that miR-214 and TRMT61A may have negative regulation. Using the miRDB database, we found that the miR-214 seed sequence is complementary to the 3′UTR region 1212–1219 of TRMT61A, indicating that TRMT61A may be a potential target of miR-214 (Figure 3A). To confirm whether the 3′UTR region of TRMT61A is a direct target of miR-214, a Dual-Luciferase Reporter experiment was performed. When the miR-214 mimic was transfected into 293T cells, the qPCR results show that the mRNA expression level of miR-214 increased significantly (*p* < 0.01) at 48 h after transfection (Figure 3B). Compared to the NC group, the miR-214 mimic can significantly reduce luciferase activity after co-transfection with the TRMT61A-3′UTR-WT vector (*p* < 0.05), while no significant difference was observed in the mutant group (TRMT61A-3′UTR-MT) (Figure 3C). After miR-214 overexpression and interference, the gene TRMT61A was significantly decreased (*p* < 0.05) and increased (*p* < 0.05), respectively (Figure 3D,E). The results show that miR-214 targets and negatively regulates TRMT61A.

## 4. Discussion

The current reports on miR-214 are mostly focused on cancer, showing that miR-214 can both inhibit the proliferation and promote the apoptosis of cervical cancer cells [13], and can also inhibit the proliferation of liver cancer cells and participate in the regulation of the cell cycle of liver cancer [14]. miR-214 gene knockout can inhibit the proliferation, migration, and invasion of gastric cancer cells [6]. At present, there is no relevant report on the role of miR-214 in chicken myoblasts. p53 is a tumor suppressor gene, which can control the initiation of the cell cycle and induce apoptosis. The p53 gene and its effector molecule, p21, play an important role in cell cycle regulation, and the transcriptional activation of p21 is positively regulated by p53. The overexpression of p53 can inhibit cell proliferation by increasing p21 levels [15]. Previous studies have shown that both p21 and p53 induce apoptosis to arrest the cell cycle [16,17]. Cai et al. regarded p21 as a marker gene for CPM proliferation [12]. Our study explored the effect of miR-214 on the proliferation of CPMs and detected the expression of p21. It was found that the overexpression of miR-214 can promote the expression of p21, while interference with miR-214 can inhibit the expression of p21. In addition, we also used flow cytometry, CCK-8, and EdU to detect the effects of the interference and overexpression of miR-214 on the proliferation of CPMs. The results show that miR-214 inhibited the proliferation of CPMs. Other studies have also shown that miR-214 can promote C2C12 cells to exit mitosis, which is similar to our results regarding the inhibition of cell proliferation [9].

During the undifferentiated stage, the transcription region of miR-214 was occupied and inhibited by the PcG protein. With the progress of differentiation, PcG began to be stripped and miR-214 began to be transcribed. After transcription, miR-214 targeted EZH2 and produced negative feedback on PcG [18]. In addition, PcG has also been shown to occupy the position of muscle development factor MYOD [19]. In undifferentiated skeletal muscle cells, although MYOD is expressed, MYOD is still occupied by PcG. PcG is then stripped from the target binding site of the MYOD after the complete myogenic program is started [20]. This evidence shows that miR-214 begins to be transcribed when differentiation begins, and the myogenic factor MYOD begins to play its role. Furthermore, MYHC, MYOD, and MYOG are the marker genes of muscle differentiation, which were used to detect the degree of differentiation of the CPMs. Huang et al. also used MYHC, MYOD, and MYOG as marker genes to detect the degree of differentiation [21]. Our results show that overexpression of miR-214 can increase the expression of MyHC, MyoD, and MyoG, while interference with miR-214 can reduce the expression of these muscle differentiation markers. At the same time, the results of the indirect immunofluorescence experiments showed that overexpression of miR-214 can significantly increase the area of myotubes (*p <* 0.01), while interference with miR-214 can significantly reduce the area of myotubes (*p <* 0.01). These results suggest that miR-214 can promote the differentiation of CPMs.

The proliferation and differentiation of CPMs are regulated by numerous microRNAs, which together regulate the proliferation and differentiation of CPMs. Although miR-214 was able to inhibit the proliferation of CPMs in the present research, other microRNAs may promote the proliferation of CPMs. Studies have shown that miR-223 can inhibit the proliferation of CPMs and can promote their differentiation [5], which is consistent with our results. There is also evidence that miR-16-5p can inhibit myoblast proliferation and can inhibit myoblast differentiation [12].

The TRMT6/TRMT61A complex can be introduced into the m^1^A structure, and almost all m^1^A are introduced by the TRMT6/TRMT61A complex. Recent studies have shown that m^1^A can destroy Watson–Crick base pairing and can participate in post-transcriptional regulation, thus affecting mRNA translation [22]. However, there are few reports about TRMT61A, and studies on its biological function are even rarer. In order to study the relationship between miR-214 and TRMT61A, we used qPCR to detect their expression in the embryonic stage and in the process of induced differentiation and found that their expression shows a negative correlation trend. Our original sequencing results also show that their expression is negatively correlated. In order to further verify whether there is a targeting relationship between miR-214 and TRMT61A, experiments such as a Dual-Luciferase Reporter assay were carried out, which confirmed the targeting relationship and negative correlation between them. After the overexpression of miR-214, the mRNA expression of its target gene, TRMT61A, was reduced, while the overexpression of miR-214 inhibited the proliferation of CPMs. Based on the above results, we can infer that TRMT61A can promote the proliferation of CPMs. In addition, there is evidence that this methyltransferase acts on gastrointestinal tumor cells through the regulation of the PI3K/AKT/mTOR and ErbB pathways, as well as promotes the proliferation of cancer cells [23].

## 5. Conclusions

In conclusion, miR-214 can both inhibit the proliferation and promote the differentiation of CPMs, and TRMT61A is a target gene of miR-214.

## Figures and Tables

**Table 1:** Gene primer sequence-related information.

Gene Name	Primer Sequences (5′ to 3′)	Product Size (bp)	Annealing Temperature (°C)	Accession Number
*TRMT61A*	F: GTGGGAAGCCATTGGACAT	266	57	XM_421386.6
	R: CTGGGCTACCTTGGTTTGAT			
*p21*	F: CCCGTAGACCACGAGCAGAT	102	61	NM_204396.1
	R: CGTCTCGGTCTCGAAGTTGA			
*MYOD*	GCTACTACACGGAATCACCAAAT	200	53	NM_204214.2
	R: CTGGGCTCCACTGTCACTCA			
*MYHC*	F: CTCCTCACGCTTTGGTAA	213	53	NM_001319304.1
	R: TGATAGTCGTATGGGTTGGT			
*MYOG*	F: CGGAGGCTGAAGAAGGTGAA	320	53	NM_204184.1
	R: CGGTCCTCTGCCTGGTCAT			
*β-actin*	F: CAGCCATCTTTCTTGGGTAT	169	60	NM_205518.1
	R: CTGTGATCTCCTTCTGCATCC			

**Table 2:** Related primer sequence information in the miR-214 quantitative experiment.

Gene Name	Primer Sequences (5′ to 3′)	Annealing Temperature (°C)
*gga-miR-214*	F: CGCGACAGCAGGCACAGAC	60
	R: AGTGCAGGGTCCGAGGTATT	
*U6*	F: GTCACTTCTGGTGGCGGTAA	60
	R: GTTCAGTAGAGGGTCAAA	
Stem loop primer	GTCGTATCCAGTGCAGGGTCCGAGGTATTCGCACTGGATACGACCTGCCT	

**Table 3:** Sequence information of miR-214 overexpression, interference, and negative control.

Sequence Name	Sequence Information
miR-214 mimic	ACAGCAGGCACAGACAGGCAG
	GCCUGUCUGUGCCUGCUGUUU
Mimic NC	UUCUCCGAACGUGUCACGUTT
	ACGUGACACGUUCGGAGAATT
miR-214 inhibitor	CUGCCUGUCUGUGCCUGCUGU
Inhibitor NC	CAGUACUUUUGUGUAGUACAA
